# Weight-bearing knee flexion angle better correlates with patient-reported outcome measures than non-weight-bearing condition in total knee arthroplasty: a three-dimensional analysis study

**DOI:** 10.1186/s12891-021-04594-x

**Published:** 2021-08-21

**Authors:** Tomofumi Kage, Hiroshi Inui, Tetsuya Tomita, Takaharu Yamazaki, Shuji Taketomi, Ryota Yamagami, Kenichi Kono, Kohei Kawaguchi, Shin Sameshima, Sakae Tanaka

**Affiliations:** 1 Department of Orthopaedic Surgery, Faculty of Medicine, The University of Tokyo, 7-3-1 Hongo, Bunkyo-ku, 113-0033 Tokyo, Japan; 2 Department of Orthopaedic Biomaterial Science, Osaka University Graduate School of Medicine, 2-2 Yamada-oka, 565-0871 Suita, Osaka Japan; 3 Department of Information Systems, Faculty of Engineering, Saitama Institute of Technology, 1690 Fusaiji, 369-0293 Fukaya, Saitama Japan

**Keywords:** Knee flexion angle, Weight-bearing, Non-weight-bearing, Patient-reported outcome measures, Three-dimensional analysis

## Abstract

**Background:**

This study aims to elucidate and compare the relationship between the knee flexion angle and patient-reported outcome measures (PROM) in both non-weight-bearing (NWB) and weight-bearing (WB) conditions.

**Methods:**

This retrospective cohort study included 61 knees (47 patients) who underwent total knee arthroplasty. The knee flexion angle was measured by three conditions: NWB in manual goniometer, NWB in fluoroscopic three-dimensional (3D) analysis and WB in the fluoroscopic 3D analysis. The PROM was evaluated by postoperative 2011 Knee Society Score (2011 KSS) and Knee injury and Osteoarthritis Outcome Score (KOOS). Correlations between the knee flexion angle and PROM was analyzed using Spearman’s correlation coefficient. Additionally, whether the angular difference between NWB and WB correlated with the PROM or not was evaluated.

**Results:**

The NWB knee flexion angle in a goniometer, NWB in 3D analysis, and WB in 3D analysis were 124.6° ± 8.4°, 118.0° ± 10.5°, and 109.5° ± 13.3°, respectively. The angular difference was 8.5° ± 12.8°. No PROM correlation existed in NWB using a goniometer. Moreover, significant positive correlations in 2011 KSS symptoms (*r* = 0.35) and 2011 KSS functional activities (*r* = 0.27) were noted in NWB using 3D analysis. Significant positive correlations existed in 2011 KSS symptoms (*r* = 0.32), 2011 KSS functional activities (*r* = 0.57), KOOS pain (*r* = 0.37), KOOS activity of daily living (ADL; *r* = 0.45), KOOS sports (*r* = 0.42), and KOOS quality of life (*r* = 0.36) in WB using 3D analysis. Significant negative correlations were noted in 2011 KSS functional activities (*r* = − 0.45), KOOS ADL (*r* = − 0.30), and KOOS sports (*r* = − 0.38) in angular difference.

**Conclusions:**

The WB knee flexion angle better correlated with PROM compared with NWB by evaluation of 3D analysis. The larger the angular difference existed between NWB and WB, the lower the PROM score.

## Background

Total knee arthroplasty (TKA) is a successful treatment for improving pain and function in patients with severe osteoarthritis (OA) [1]. However, approximately 20 % of TKA patients are not satisfied with surgery outcomes [2–4]. The knee flexion angle is considered as one major parameter of all the postoperative clinical outcomes [2, 5]. Various studies have currently reported the relationship between postoperative knee flexion angle and patient-reported outcome measures (PROM). On the one hand, most of the reports described the positive relationship between knee flexion angle and PROM [2, 5–7]. On the other hand, several reports described the weak or modest relationship between the knee flexion angle and PROM [8–10]. Thus, whether the knee flexion angle affects the PROM or not is controversial in some way. Two reasons are possible factors. First, the knee flexion angle measurement was performed using the manual goniometer in most studies. Moreover, the measurement using a manual goniometer is reported to be likely inaccurate [11, 12]. More accurate methods such as the three-dimensional (3D) measurement would have the different results. Second, the angle measurement was usually performed under non-weight-bearing (NWB) conditions [13, 14]. Most of the activities of daily living (e.g., squatting, climbing up and down the stairs, or rising from a chair) and recreational activities (e.g., gardening) require performing motion under weight-bearing (WB) condition. The event that the same patient cannot flex the knee deeply in WB condition although able to do so in NWB condition is sometimes experienced in clinical practice. Hence, the NWB knee flexion angle measurement only may be inadequate to investigate the relationship with PROM. The WB knee flexion angle may reflect the PROM more compared with NWB, and the measurement of knee motion in WB fashion may be a superior method to assess functional capabilities [15].

However, which goniometer measurement or 3D measurement reflects PROM more is unknown. Similarly, which NWB or WB condition reflects PROM more is not known. Additionally, whether the angular difference of the knee flexion angle between NWB and WB conditions in the same patient influence the PROM or not is unclear. Thus, the first purpose of this study was to elucidate which goniometer or 3D measurement reflected the PROM more and which NWB or WB condition affected the PROM more using a 3D measurement. The second purpose was to elucidate whether the angular difference of the knee flexion angle between NWB and WB influenced the PROM or not.

## Methods

This study was approved by our institutional review board (number 10,462-(1)), and all patients provided written informed consent. This retrospective cohort study was based on 61 knees in 47 patients who underwent TKA at our institution from November 2014 to May 2019. The inclusion criteria were providing consent for fluoroscopic evaluation, patients with no deficit of PROM scores, varus knee OA, Kellgren–Lawrence OA grade III or IV and primary bicruciate-stabilized TKA (The Journey II BCS; Smith & Nephew, Memphis, TN, USA). Conversely, the exclusion criteria were rheumatoid arthritis, post-traumatic arthritis, valgus knee OA, revision TKA and TKA with an implant of other types. Table 1 shows patient demographics. These demographic data were expressed as mean ± standard deviation. Using full-length standing radiographic images, the hip–knee–ankle angle was measured. The timing of fluoroscopic survey was 13.5 ± 7.8 (range, 6–36) months after surgery. All patients were operated on by the same surgical team and a highly experienced surgeon (H.I.) took part in all the procedures as either the chief surgeon or the first assistant. The surgery was performed using the image-free navigation system (Precision N, Stryker Orthopedics, Mahwah, NJ, USA).

**Table 1:** Patient demographics

Number of knees	61
Number of patients	47
Gender (female/male)	40 / 7
Age (years)	74.8 ± 7.2
Body height (cm)	155.0 ± 6.5
Body weight (kg)	64.6 ± 10.6
Body mass index (kg/m^2^)	26.9 ± 4.0
Preoperative hip–knee–ankle angle (°)	167.7 ± 5.6
Postoperative hip–knee–ankle angle (°)	179.1 ± 2.4
Follow-up (months)	13.5 ± 7.8

### Surgical procedure

All the patients underwent a paramedian approach, and the patella was not everted. The coronal alignment of the distal femur and the proximal tibia were cut to be perpendicular to the mechanical axis. The sagittal alignment of the distal femur was aligned at 4° of flexion to prevent anterior femoral notching [16], and that of the proximal tibia was aligned 3° of the posterior slope according to manufacturer’s recommendations. Soft-tissue balancing was performed, and the extension and flexion gaps were measured using balancer devices [17]. The amount of the posterior femur resection was adjusted to equalize the extension and flexion gaps of the medial compartment [18]. The femoral rotational axis was aligned to be parallel to the surgical epicondylar axis. The rotational position of the tibial component was obtained by placing the knee through a full range of motion while allowing the tibial trial component to orient itself into a matched position relative to the femoral component, thereby reducing rotational mismatch of the components [19].

### The measurement of the knee flexion angle

**Fig. 1:**
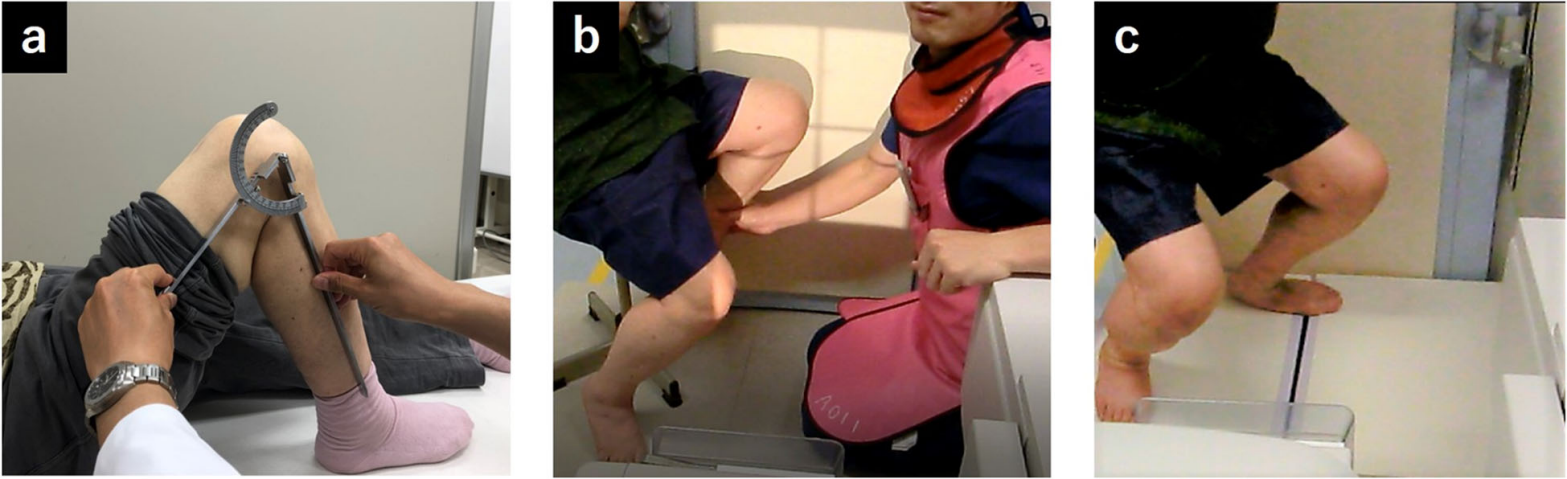
(**a**) The non-weight-bearing (NWB) knee flexion angle was measured using a goniometer. (**b**) The NWB knee flexion angle was measured using a fluoroscopic three-dimensional (3D) analysis. (**c**) The weight-bearing (WB) knee flexion angle was measured using fluoroscopic 3D analysis

First, the knee flexion angle using a goniometer was measured. The patient was placed in a supine position with maximal active-assisted knee bending without causing pain. The investigator measured the knee flexion angle using a goniometer (Fig. 1a) and the angle was measured with 5° increments. The goniometer had two arms with lengths of 50 cm. During measurement, the center of the goniometer was kept on the joint center with the distal arm pointing to be the lateral malleolus and the proximal arm pointing to the greater trochanter.

Second, each patient performed two different deep knee bends during single-view fluoroscopic monitoring in the sagittal plane to measure the knee flexion angle using 3D measurement. The first was active-assisted deep knee bending (NWB; Fig. 1b) and the second was squatting (WB; Fig. 1c). The active-assisted deep knee bending was assisted by an operator without causing pain. Both knee bends were performed to maximum flexion at a natural pace according to a previously described method [20]. The participants practiced the motions several times before recording. The sequential motion was recorded as digital radiographic images (1,024 × 1,024 × 12 bits/pixel, 7.5 Hz serial spot images in a Digital Imaging and Communications in Medicine format), using a 17 inch flat-panel detector system (ZEXIRA DREX-ZX80; Toshiba, Tokyo, Japan). All images were obtained using dynamic- range compression to acquire edge-enhanced images. A two-dimensional (2D) to three-dimensional (3D) registration technique was used to estimate the spatial position and orientation of the femoral and tibial components [21–23]. This technique was based on a contour-based registration algorithm with 2D single-view fluoroscopic images and 3D computer-aided design models. The estimated accuracy of the relative motion between the femoral and tibial components was ≤ 0.4 mm and ≤ 0.5° for translations and rotations, respectively [22]. A local coordinate system of the component was set according to a previously described method. The origin of the femoral component was located at the center of gravity of the component, and the origin of the tibial component was located at the center of the surface of the tibial tray [20, 22]. The knee flexion angle was expressed according to the joint’s rotational conventional method described by Grood and Suntay [24]. Of all images, the maximum knee flexion angle was adopted as the knee flexion angle. Accordingly, the knee flexion angles in WB and NWB conditions using 3D measurement were calculated, respectively.

Additionally, the angular difference was calculated using the knee flexion angle in the WB and NWB conditions measured by 3D analysis. The angular difference was defined as the angle in the NWB condition minus the angle in the WB condition. The positive values mean that the angle in the NWB condition is larger than that in the WB condition.

### Patient-reported outcome measures (PROM)

Two clinical outcome measures were evaluated in the PROM. The first outcome measure was symptoms, satisfaction, expectation, and functional activities of the 2011 Knee Society Score (2011 KSS) [25]. The highest scores of the four subscales, which represent no knee problems, are 25, 40, 15, and 100 points, respectively. The second outcome measure was pain, symptoms, and activities of daily living (ADL), sports, and quality of life (QOL) of the Knee injury and Osteoarthritis Outcome Score (KOOS), which is a valid and reliable outcome measure for TKA patients [26–28]. The highest scores of the five subscales, which represent no knee problems, are 100 points. The 2011 KSS score was evaluated at 1 year postoperatively, and the KOOS score was evaluated at the timing of the fluoroscopic survey.

Finally, the correlations between the four following knee flexion angles (NWB using a goniometer, NWB using 3D measurement, WB using 3D measurement, and the angular difference using 3D measurement) and the two PROMs (2011 KSS and KOOS) were evaluated.

### Statistical analysis

Statistical analyses were performed using Statistical Package for the Social Sciences (version 25, IBM Corporation, Armonk, NY, USA). Spearman’s correlation coefficient was used to analyze the correlation between the knee flexion angle and the PROM. A linear regression analysis was also conducted. A power analysis was performed prior to the start of this study using G*Power (version 3.1.9.4, Heinrich Heine University, Düsseldorf, Germany) [29]. Using an effect size of 0.35, an estimated sample size of 61 was required (1 − β = 0.80, α = 0.05). P values of < 0.05 were considered statistically significant.

The reliability of 3D measurement was evaluated by intraclass and interclass correlation coefficients. The measurement was performed twice by one surgeon and once by another, on 10 knees randomly selected from the study group. The intraclass and interclass coefficient values were both 0.99, indicating excellent reliability.

## Results

### Knee flexion angle

Table 2 shows the knee flexion angle under four settings. These data were expressed as mean ± standard deviation.

**Table 2:** Knee flexion angle

	Degrees (mean ± SD)
NWB (goniometer)	124.6 ± 8.4
NWB (3D)	118.0 ± 10.5
WB (3D)	109.5 ± 13.3
Angular difference (NWB – WB: 3D)	8.5 ± 12.8

*NWB* non-weight-bearing; *WB* weight-bearing; *3D* three-dimension; *SD* standard deviation

### Correlation between the knee flexion angle and PROM

**Fig. 2:**
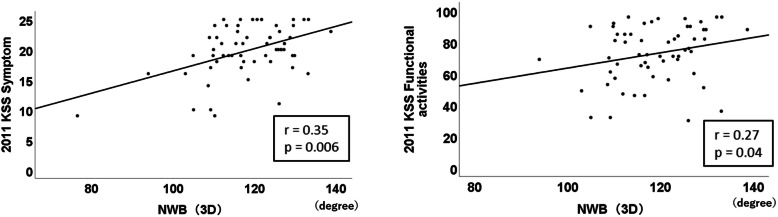
Scatter diagram of significantly correlated subscales with the non-weight-bearing (NWB) flexion angle using three-dimensional (3D) measurement

**Fig. 3:**
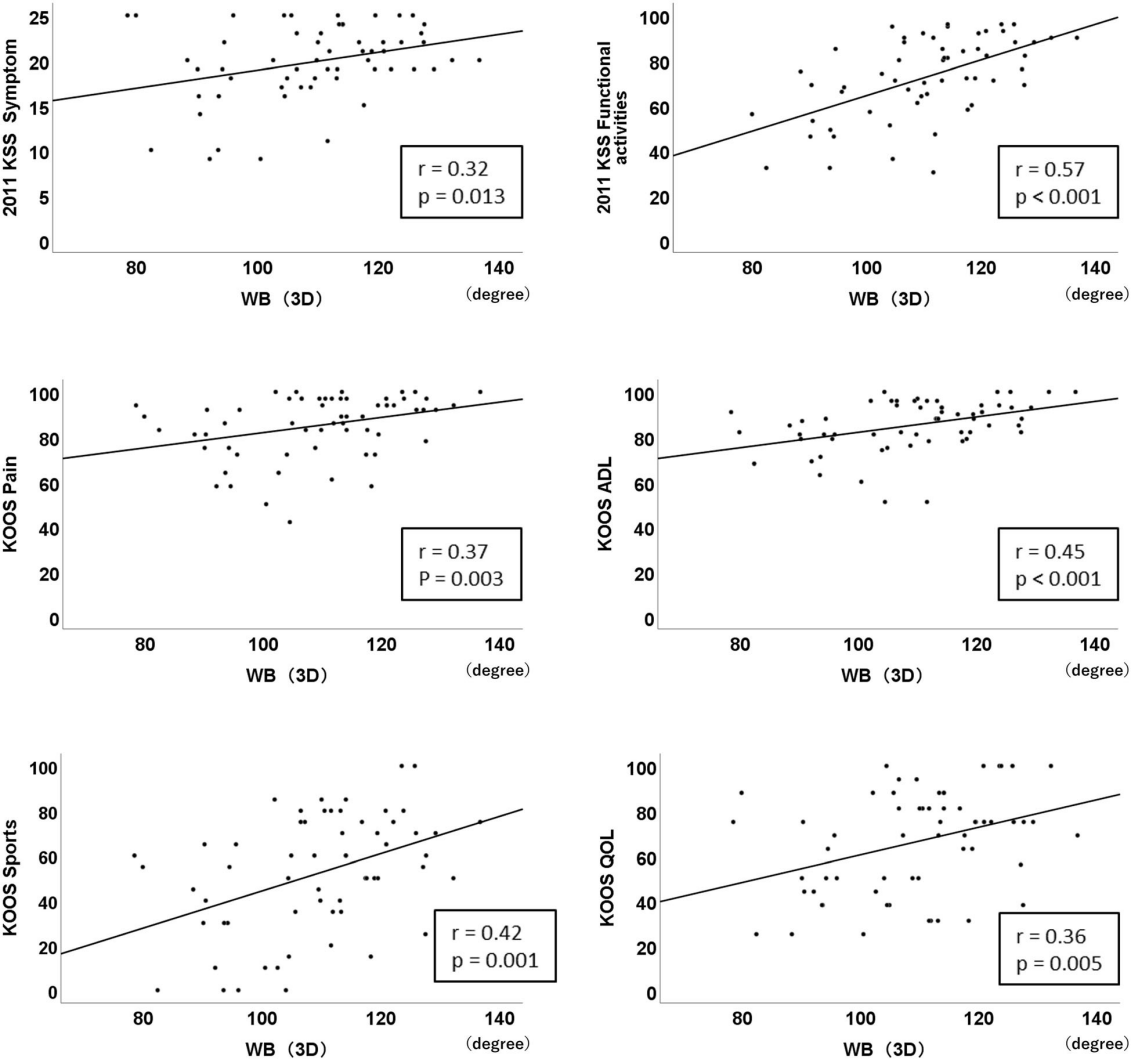
Scatter diagram of significantly correlated subscales with the weight-bearing (WB) flexion angle using three-dimensional (3D) measurement

**Fig. 4:**
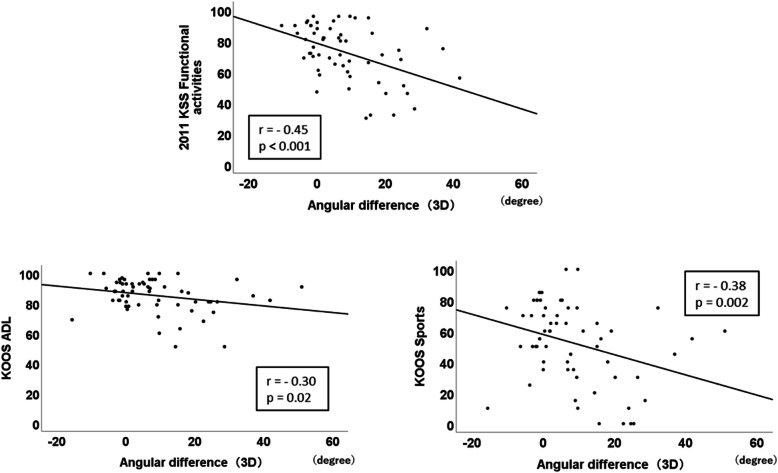
Scatter diagram of significantly correlated subscales with the angular difference between non-weight-bearing (NWB) and weight-bearing (WB) using three-dimensional (3D) measurement

In the NWB knee flexion angle using a goniometer, no significant correlation existed between the angle and PROM (Table 3). However, in the NWB knee flexion angle using a 3D measurement, significant positive correlations with 2011 KSS symptoms (*r* = 0.35, *p* = 0.006) and 2011 KSS functional activities (*r* = 0.27, *p* = 0.04; Table 3) existed. Figure 2 shows the significant parts of the subscales and scatter diagram. In the WB knee flexion angle using 3D measurement, significant positive correlations existed with 2011 KSS symptoms (*r* = 0.32, *p* = 0.013), 2011 KSS functional activities (*r* = 0.57, *p* < 0.001), KOOS pain (*r* = 0.37, *p* = 0.003), KOOS ADL (*r* = 0.45, *p* < 0.001), KOOS sports (*r* = 0.42, *p* = 0.001), and KOOS QOL (*r* = 0.36, *p* = 0.005; Table 3). Figure 3 shows the significant parts of the subscales and scatter diagram. In the angular difference between NWB and WB, significant negative correlations with 2011 KSS functional activities (*r* = − 0.45, *p* = 0.02), KOOS ADL (*r* = − 0.30, *p* = 0.02), and KOOS sports (*r* = − 0.38, *p* = 0.002; Table 3) existed. Figure 4 shows the significant parts of the subscales and scatter diagram.

**Table 3:** Correlation coefficients between the knee flexion angle and patient-reported outcome measures

	2011 KSS				KOOS				
Knee flexion angle	Symptoms	Satisfaction	Expectation	Functionalactivities	Pain	Symptoms	ADL	Sports	QOL
NWB (goniometer)	0.22	0.02	0.15	0.11	0.01	0.07	0.09	-0.03	-0.01
NWB (3D)	0.35**	0.17	-0.03	0.27*	0.22	0.21	0.23	0.08	0.18
WB (3D)	0.32*	0.20	-0.07	0.57***	0.37**	0.15	0.45***	0.42**	0.36**
Angular difference(NWB – WB: 3D)	-0.07	-0.07	0.06	-0.45***	-0.18	0.02	-0.30*	-0.38**	-0.23

*NWB* non-weight-bearing; *WB* weight-bearing; *3D* three-dimension; *KSS* Knee Society Score; *KOOS* Knee injury and Osteoarthritis Outcome Score; *ADL* Activities of daily living; *QOL* Quality of life

*Correlation is statistically significant (*p* < 0.05)

**Correlation is statistically significant (*p* < 0.01)

***Correlation is statistically significant (*p* < 0.001)

## Discussion

The first important findings in this study were that the 3D measurement of the knee flexion angle better correlated with PROM compared with the goniometer measurement and the WB knee flexion angle better correlated with PROM compared with the NWB condition. The second important finding was that the angular difference between NWB and WB correlated with PROM. To our best knowledge, this paper is the first report to discuss the positive correlation between WB knee flexion angle and PROM using a precise 3D measurement. Additionally, the correlation between angular difference and PROM was reported.

The NWB knee flexion angle using 3D measurement correlated with 2011 KSS symptoms and 2011 KSS functional activities, although the angle using goniometer measurement did not correlate with PROM at all. This result suggests that the 3D measurement may reflect the PROM more than goniometer measurement. The difference of results between the 3D and goniometer measurement may be attributed to the difference in measurement method. The 3D measurement is reported to be precise [22], whereas the goniometer measurement is manual. A recent report described that the standard deviation of the long arm goniometer measurement was 4.9° [12]. Additionally, goniometer measurement was performed with 5° increments. If the goniometer method is more accurate, the knee flexion angle might correlate with PROM. The measurement of the knee flexion angle using 3D is desirable for precise assessment. However, the manual goniometer is widely used in clinical practice where the 3D measurement is not set up. Thus, the surgeons should keep in mind the potential inaccuracy of the manual goniometer. We are now trying an alternative measurement method using roentgen measurement to apply easily in general hospitals because the 3D measurement cannot be used generally in the hospitals.

The results of the present study showed that the knee flexion angle in the WB condition correlated more with PROM than in the NWB condition in precise 3D measurement. This result suggests that the assessment of the WB angle may be more reasonable and appropriate than the NWB to evaluate the relationship between the angle and PROM. The results of this study were compatible with a previous study by Song et al. [30] reporting that active flexion in WB better correlates with the functional outcomes of TKA than passive flexion. However, the study by Song et al. [30] measured the angle using a goniometer. Therefore, the results may lack accuracy because of the measurement method [11, 31]. This study confirmed the superiority of the evaluation of WB condition by precise 3D measurement. Therefore, this is a strong point of the present study. One possible reason why the results of this study showed more correlation in WB than in NWB may be attributed to the fact that a substantial portion of ADL are performed under WB condition [15]. A previous report revealed that the activities requiring WB knee flexion (e.g., climbing up or down a flight of stairs, getting into and out of a car, and squatting) was very important and distressing for patients who have undergone TKA [32]. Thus, the ability to squat may be required in the present study. Based on the results of this study, to appropriately evaluate the relationship with PROM, the surgeons should evaluate not only the NWB knee flexion angle but also the WB knee flexion angle.

The current findings demonstrated that the satisfaction score did not correlate with the knee flexion angle. The finding was consistent with the report from the United States by Devers et al. [8]. This indicates that the relationship between satisfaction and knee flexion angle may be similar in Asian and Western countries. Regarding the functional score, the current findings showed that the knee flexion angle by 3D measurement significantly correlated with 2011 KSS functional activities. Park et al. also reported that knees with a maximum flexion of more than 135° had a better functional Western Ontario McMasters Universities Osteoarthritis Index score in Asian patients [9]. Conversely, Meneghini et al. reported that obtaining flexion greater than 125° did not offer a benefit in overall knee function [10]. These results imply that greater knee flexion may lead to higher functional score especially in Asian patients who require high flexion activities.

Significant negative correlations were found between angular difference and PROM in 2011 KSS functional activities, KOOS ADL, and KOOS sports. This result suggested that the larger the angular difference existed between NWB and WB, the lower the PROM score. The present study showed that the WB knee flexion angle was 8.5° ± 12.8° smaller than the NWB knee flexion angle. This result was consistent with the study by Dennis et al. [15] reporting that the WB knee flexion angle was smaller than the NWB in TKA knees. However, the relationship between the angular difference and PROM was not described in their study [15]. This study confirmed the relationship between the angular difference and PROM, which may be meaningful. It is difficult to explain why some patients have larger angular difference despite some patients acquiring as much WB flexion angle compared with that in the NWB condition. Muscle strength may be related although the muscle strength of the lower extremities could not be evaluated. Dennis et al. described that the angular decrease in WB compared with NWB was presumably resulted from the complex interaction of dynamic muscle forces [15]. Based on the results of this study, acquiring as much WB flexion angle compared with that in the NWB condition may potentially lead to higher PROM in terms of postoperative rehabilitation. In order to gain WB knee flexion angle, the closed kinetic chain exercises (e.g., squatting and sit-to-stand exercise with/without handrail) is considered to be necessary. However, whether the rehabilitation emphasized to obtain more WB knee flexion angle is effective or not is still unknown. Hence, further case–control and interventional research are necessary. When it comes to surgery, friendly handling of soft tissues including muscles may be needed during surgery. Additionally, surgeons should recognize whether the angular difference exists or not by checking not only the NWB flexion angle but also the WB flexion angle.

The clinical relevance of this study was that the WB knee flexion angle was better correlated with PROM compared with the NWB knee flexion angle. Thus, surgeons must recognize the importance of the WB knee flexion angle and should evaluate not only the knee flexion angle in the NWB condition but also that in the WB condition.

However, several limitations in this study should be acknowledged. First, the single implant design was evaluated in this study. Other types of TKA implant might present other results. Second, the alignment of implants was not evaluated in this study. Third, the knee flexion angle and PROM were only evaluated postoperatively. The preoperative angle and PROM may have influenced the postoperative results. Fourth, the timing of the evaluation of the KOOS score was relatively ranged among patients because the timing of the KOOS score depended on the timing of the fluoroscopic survey. Fifth, the knee position during the knee flexion differed in the same NWB condition between the goniometer and fluoroscopic 3D measurement. In the NWB condition using a goniometer, the knee position was performed in a usual clinical manner. However, in the NWB condition using fluoroscopic 3D measurement, the knee position when sitting on the chair was applied to suitably capture the knee in fluoroscopy. Sixth, the measurement method was different between the goniometer and 3D measurement. The flexion angle using a goniometer was measured between the femoral and tibial bones, whereas the flexion angle using 3D analysis was measured between the femoral and tibial components. Seventh, the reliability and reproducibility of the goniometer measurement were not evaluated in this study. We are planning to investigate those in future studies. Lastly, the muscle strength of the lower extremities was not evaluated. The muscle strength may influence the knee flexion angle, particularly in the WB condition.

## Conclusions

The WB knee flexion angle better correlated with PROM than the NWB knee flexion angle by the evaluation of precise 3D analysis. Additionally, the larger the angular difference existed between NWB and WB conditions, the lower the PROM score.

## Data Availability

The datasets generated and analyzed during the current study are not publicly available due to privacy concern of participants but are available from the corresponding author on reasonable request.

## Abbreviations

TKA: Total knee arthroplasty
OA: Osteoarthritis
PROM: Patient-reported outcome measures
3D: Three-dimensional
NWB: Non-weight-bearing
WB: Weight-bearing
2D: Two-dimensional
KSS: Knee Society Score
ADL: Activities of daily living
QOL: Quality of life
KOOS: Knee injury and Osteoarthritis Outcome Score
